# Multimodal decoding and congruent sensory information enhance reaching performance in subjects with cervical spinal cord injury

**DOI:** 10.3389/fnins.2014.00123

**Published:** 2014-05-23

**Authors:** Elaine A. Corbett, Nicholas A. Sachs, Konrad P. Körding, Eric J. Perreault

**Affiliations:** ^1^Sensory Motor Performance Program, Rehabilitation Institute of ChicagoChicago, IL, USA; ^2^Department of Physical Medicine and Rehabilitation, Northwestern UniversityChicago, IL, USA; ^3^Melbourne School of Psychological Sciences, University of MelbourneParkville, VIC, Australia; ^4^Department of Biomedical Engineering, Northwestern UniversityEvanston, IL, USA; ^5^Department of Physiology, Northwestern UniversityChicago, IL, USA

**Keywords:** eye-tracking, electromyography, spinal cord injury, Kalman filter, proprioceptive feedback

## Abstract

Cervical spinal cord injury (SCI) paralyzes muscles of the hand and arm, making it difficult to perform activities of daily living. Restoring the ability to reach can dramatically improve quality of life for people with cervical SCI. Any reaching system requires a user interface to decode parameters of an intended reach, such as trajectory and target. A challenge in developing such decoders is that often few physiological signals related to the intended reach remain under voluntary control, especially in patients with high cervical injuries. Furthermore, the decoding problem changes when the user is controlling the motion of their limb, as opposed to an external device. The purpose of this study was to investigate the benefits of combining disparate signal sources to control reach in people with a range of impairments, and to consider the effect of two feedback approaches. Subjects with cervical SCI performed robot-assisted reaching, controlling trajectories with either shoulder electromyograms (EMGs) or EMGs combined with gaze. We then evaluated how reaching performance was influenced by task-related sensory feedback, testing the EMG-only decoder in two conditions. The first involved moving the arm with the robot, providing congruent sensory feedback through their remaining sense of proprioception. In the second, the subjects moved the robot without the arm attached, as in applications that control external devices. We found that the multimodal-decoding algorithm worked well for all subjects, enabling them to perform straight, accurate reaches. The inclusion of gaze information, used to estimate target location, was especially important for the most impaired subjects. In the absence of gaze information, congruent sensory feedback improved performance. These results highlight the importance of proprioceptive feedback, and suggest that multi-modal decoders are likely to be most beneficial for highly impaired subjects and in tasks where such feedback is unavailable.

## Introduction

Injuries to the cervical spinal cord can be devastating, resulting in lost function in both the upper and lower limbs. Many people with such injuries consider the restoration of hand and arm function to be of highest importance for improving their quality of life (Anderson, 2004; Collinger et al., 2013). People with high tetraplegia—injuries at the fourth cervical level (C4) or above—may have no movement in the arm except for possibly shoulder shrug through upper trapezius activity. For these individuals, simple every-day tasks such as feeding and grooming cannot be achieved without assistance. Consequently, methods to improve reaching and grasping could greatly increase the level of independence for this population. One of the major difficulties associated with developing such assistive devices is the limited set of physiological signals available for use in a control interface. Furthermore, sensory feedback of the reaching movement may be vital for control, and is often impaired or absent in these individuals. As more complex systems are being developed that can provide control of continuous reach trajectories to people with high tetraplegia (Crema et al., 2011; Hart et al., 2011; Cooman and Kirsch, 2012; Schearer et al., 2013), finding appropriate signal sources and developing intuitive user interfaces is an even greater challenge.

Many approaches for inferring an intended reach trajectory rely on neural signals, of which electromyograms (EMGs) are an attractive option when a non-invasive or minimally invasive approach is desired (Kilgore et al., 2008). However, when the set of available muscles is extremely limited or unrelated to the intended movements, control can be difficult and unintuitive (Williams and Kirsch, 2008). Brain-machine interfaces (BMIs) have the potential to provide more natural control (Collinger et al., 2012; Ethier et al., 2012; Hochberg et al., 2012), although most BMIs that have successfully controlled reach involve invasive cortical recordings, a technology that is currently inaccessible in most clinical situations. Combining information from disparate sources has been proposed as a solution when there are few signals accessible (Batista et al., 2008; Pfurtscheller et al., 2010; Leeb et al., 2011; Corbett et al., 2013a; Novak et al., 2013; Kirchner et al., 2014). As the set of usable signals from each individual may be different, it is important to be able take advantage of all the useful channels available. To gain an understanding of how the benefits afforded by different combinations of signal sources are influenced by impairment level, interface approaches must be tested in users with a variety of needs and abilities.

The feedback provided to the user is also critical when controlling trajectories. The type of feedback can vary depending on the function of the interface and the needs of the user. External robotic arms can enable people to interact with their environment (Hochberg et al., 2012), typically providing only visual feedback of the robot during control. Recent research promises to enhance control by artificially providing additional feedback through electrical (Dhillon and Horch, 2005; London et al., 2008; Rossini et al., 2010; Tan et al., 2013) or optogenetic (Gilja et al., 2011) stimulation. However, a subset of users may be able to take advantage of at least some natural proprioceptive information if their arm is moved with the assistive device. This could be achieved by mechanically moving the hand and arm with a robotic exoskeleton (Cavallaro et al., 2006), or using functional electrical stimulation (FES) to stimulate the motor nerves and re-animate paralyzed muscle (Hart et al., 1998; Kilgore et al., 2008; Schearer et al., 2013). Proprioception is critical in normal motor control (Sainburg et al., 1995), and studies suggest that it can also enhance BMI performance in unimpaired monkeys (Suminski et al., 2010) and humans (Ramos-Murguialday et al., 2012). It is still unclear how assisted reaching in paralyzed individuals is affected by whether they are controlling movement of their arm vs. an external device.

The objective of this study was to investigate how the control of reach trajectories in individuals with cervical SCI was affected under various decoding conditions, by testing two critical aspects of the interface. We evaluated the utility of combining disparate signal sources to enhance trajectory control, and also compared two different feedback approaches. The participants had a wide range of impairment levels; some had substantial control of the proximal arm muscles, while others had little or no ability to move the arm. We tested their performance using two decoders—one combining gaze and EMG and another with EMG alone—in a robot-assisted reaching paradigm that we had previously developed (Corbett et al., 2013a). We also evaluated how reaching performance was influenced by task-related sensory feedback by testing the decoder using EMG alone under two conditions—comparing remote control of the robot to that when the robot moved the arm in the task. By evaluating these tasks in people with a variety of injury characteristics we could examine the benefits of the different assisted reaching approaches with respect to their level of impairment. Portions of this work were presented at the 6th International IEEE EMBS Conference on Neural Engineering (Corbett et al., 2013b).

## Materials and methods

To establish the utility of the multimodal decoder, combining gaze and EMG, we compared its performance to a decoder that used EMG alone and one combining EMG with perfect target information. While perfect target information would be unlikely to be available in a practical setting, this condition was useful for comparison, serving as a best-case scenario for the paradigm. These comparisons were performed in subjects with a range of injury levels, so that the benefits afforded by sensor fusion for different impairment levels could be assessed. Additionally, to assess the importance of providing the subjects with congruent sensory feedback of the task we compared the assisted reaching task to a remote control paradigm with the EMG-alone decoder. This was performed by a subset of subjects who could activate sufficient muscles to make control with EMG alone viable. The decoding algorithms have been previously described in detail (Corbett et al., 2012, 2013a, 2014); here we outline the intuition behind them before describing the experiments, which are the main contribution of the present work.

### Decoding algorithms

We used a Bayesian approach to combining signal sources, taking into account the uncertainty inherent in the predictions of the various models. The decoder using EMG alone was a generic Kalman filter (KF) (Kalman, 1960; Wu et al., 2006). The state vector that we were trying to estimate consisted of the reach kinematics. At each time-step the KF propagated a prior estimate of the current state from the previous state posterior estimate using a linear trajectory model that described the probabilistic evolution of the state. This prior estimate was then updated using that time-step's observation—features from the corresponding window of EMG—through a linear observation model, resulting in the current posterior state estimate. For the KF, trained using reaches to a set of targets, the trajectory model biased the movement toward an “average target,” while movements in other directions could be generated through the observations (the subject's EMG).

We created a directional trajectory model by inserting the target position into the state vector (Kemere and Meng, 2005; Mulliken et al., 2008). With perfect knowledge of the target we called this model the KFT. In this case the trajectory model biased the movement toward the target, thus requiring less directional change through the user's EMG in the observation update. This inclusion of the target into the trajectory model also allowed for a more stereotyped model of the reach, where the hand would speed up when the target was distant and slow down when it was close (Corbett et al., 2012).

When obtaining target estimates from gaze we had to account for multiple potential targets, as people may also look at other locations before initiating a reach. To achieve this we used mixture of KFTs (mKFT), where we initiated an instance of the KFT for each potential target and weighted them probabilistically. The weights were proportional to a prior probability for each target that we obtained from the gaze data, and the likelihood of the observations (EMG) for each model. Therefore, as the reach progressed and more EMG information was integrated the decoder output converged to the most likely of the possible trajectories. The signal sources used in each of the algorithms are summarized in Table 1.

**Table 1:** **Decoders tested and the corresponding signal sources**.

**Algorithm**	**Signal Sources**
Kalman filter (KF)	EMG
Kalman filter with target (KFT)	EMG + target location
Mixture of KFTs (mKFT)	EMG + target estimates from gaze

### Subjects

Eight subjects with tetraplegia participated in this study. Each subject provided informed consent to the protocol, which was approved by Northwestern University's Institutional Review Board. Before commencing the experiment, we asked the subject a few basic questions to ensure safety in the experiment and to establish the number of years since his/her injury. We asked the subjects to perform shoulder flexion voluntarily, and measured the angle achieved. The subjects were separated into two groups. Group 1 consisted of the subjects who could perform more than 5° of shoulder flexion in their right arm using the deltoid muscle; subjects who had little or no voluntary ability to perform this movement made up Group 2. Shoulder flexion was the degree of freedom that best reflected the subjects' ability to perform the task (see description in Section Experimental setup). Group 1 included subjects who participated in the decoder comparison experiments (Group 1a) and the remote control experiments (Group 1b). There was substantial overlap between these groups but they were not identical due to subject availability (see Table 2). Group 2 only participated in the decoder comparison experiments.

**Table 2:** **Subject details**.

	**Injury**	**Voluntary shoulder flexion (degrees)**	**Age**	**Years since injury**	**Group**	**Notes**
1	C5/C6 incomplete	55	48	32	1a, 1b	Possible “lazy eye”
2	C5/C6 complete	180	47	26	1a, 1b	
3	C5 complete	30	41	27	1a, 1b	
4	C3/C4 incomplete	40	79	6	1a	Unable to obtain eye-tracking data
5	C4/C5 incomplete	<5	26	2	2	
6	C4 complete	0	19	2	2	No deltoid activity
7	C6/C7 complete	180	34	4	1b	
8	C4/C5 dislocation	90	34	2	1b	

### Experimental setup

To generate reaching movements we used a robotic system that served as an assisted reaching prosthesis. Each subject was seated in his/her own wheelchair during the experiments. For the experiments in which the subject's arm was moved through the reaches, his/her right arm was supported against gravity by an elevating mobile arm support (JAECO Orthopedic MASEAL, Hot Springs, AR), while he/she wore a wrist splint that was attached to the handle of a 3 degree-of-freedom robot (HapticMaster; Moog FCS, the Netherlands). A magnet attachment was designed to release if excessive forces were applied at the hand. The velocities predicted by the decoders were used to position the robot handle, enabling a clear comparison of performance issues related to decoders and signal sources.

**Figure 1:**
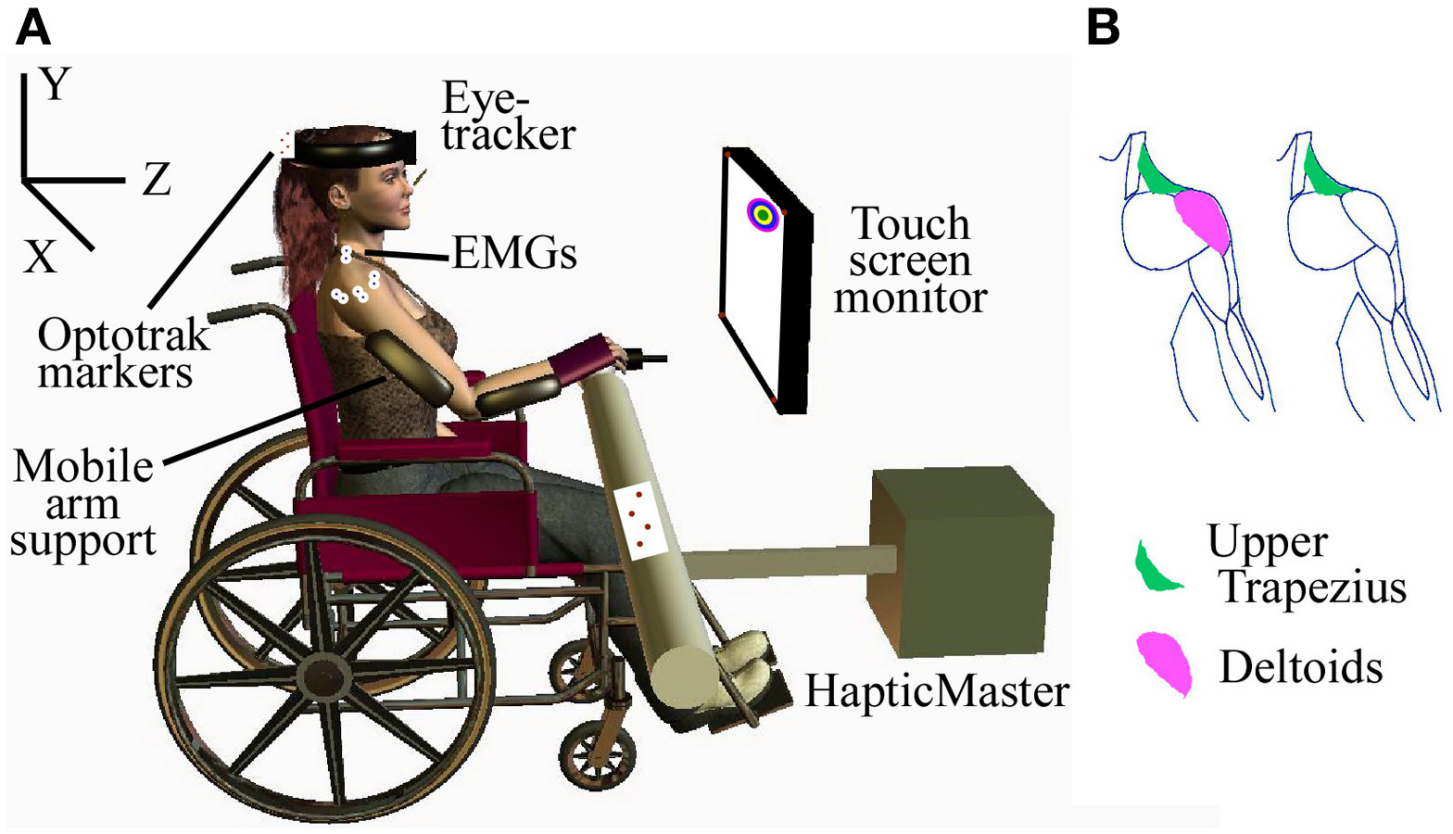
**Experimental setup**. **(A)** Subject with SCI performing assisted-reaching task with multimodal decoder; **(B)** EMGs recorded in the subjects, based on the muscles that could be voluntarily activated.

All experiments involved a reaching task, either with (Assessing the influence of impairment on decoder performance) or without (Assessing the influence of proprioceptive feedback on decoding performance) the subject's arm attached to the robot. The goal of the task was to move the robot to a target on a touch-screen monitor (Planar PT19, Beaverton, OR) in front of the subject (Figure 1A). A spring-loaded stylus was attached to the robot end-effector, and used to detect contact with the target. The monitor and HapticMaster positions were recorded using an Optotrak motion analysis system (Northern Digital Inc., Canada) so that positions on the monitors could be transformed into the HapticMaster coordinate system. We recorded eye movements with an EYETRAC-6 head mounted eye tracker (Applied Science Laboratories, Bedford, MA), whose position was also monitored with the Optotrak. The position of the eye was digitized relative to the eye-tracker before its use, so that the gaze data could be projected onto the screen and transformed into the appropriate coordinate systems. All signals were recorded simultaneously and processed at 60 Hz, so as to generate a real-time velocity command signal to control the robot.

Consistent with our previous experiments in able-bodied subjects, we recorded EMGs from the three heads of the deltoid and the upper trapezius from the subjects who could voluntarily activate those muscles, and just the upper trapezius from one subject who had no voluntary control of the deltoid (Figure 1B). The EMG signals were amplified and band-pass filtered between 10 and 1000 Hz using a Bortec AMT-8 (Bortec Biomedical Ltd., Canada), anti-alias filtered using 5th order Bessel filters with a cut-off frequency of 500 Hz, and sampled at 2400 Hz. Features were extracted from a 16.6 ms window of each EMG channel for use as observations in each of the decoders. The square-root transformed RMS and number of zero-crossings were selected as amplitude and frequency related features, respectively.

### Protocols

#### Assessing the influence of impairment on decoder performance

**Figure 2:**
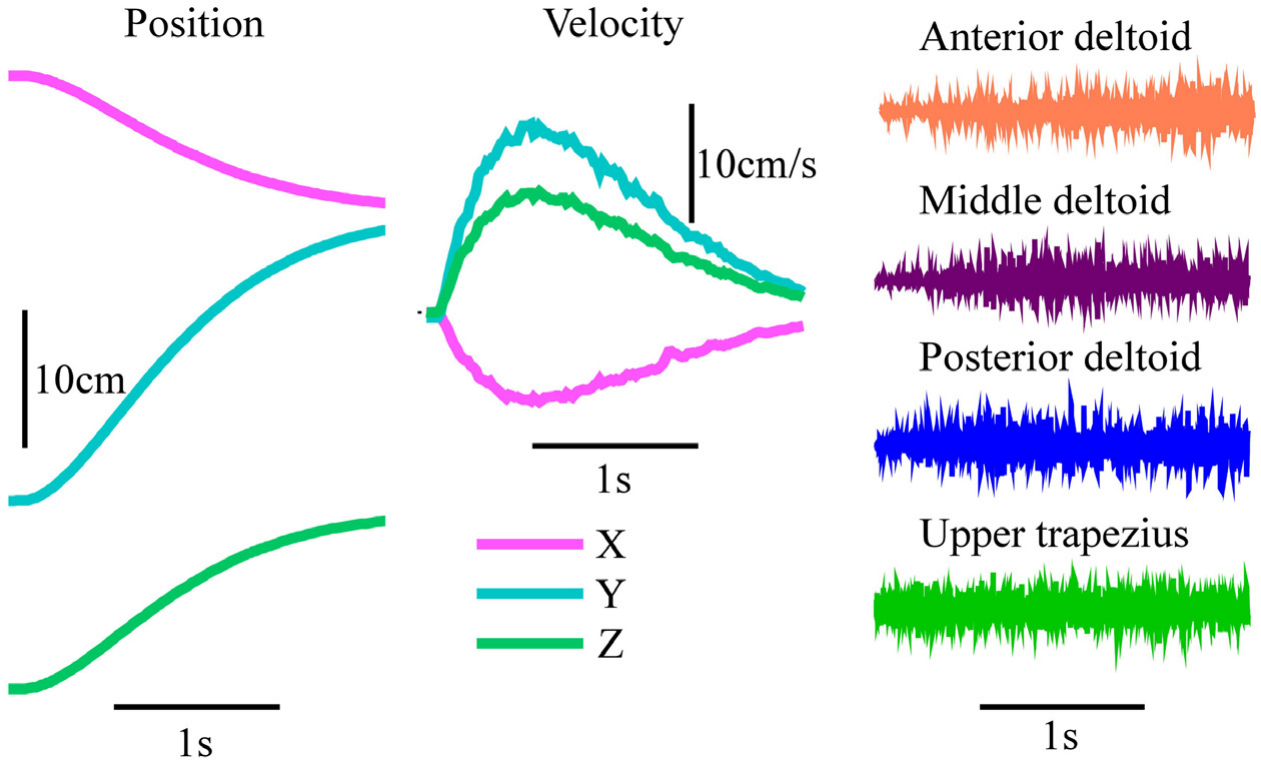
**Example training reach and EMG**. Automatically generated robot kinematics and EMG signals produced by one subject from Group 1 as they assisted the reach during the training protocol.

The goal of the first set of experiments was to establish the utility of combining gaze with EMG, compared to decoding with EMG alone, with subjects spanning a range of needs and abilities. For these experiments the robot moved the subjects' arms along with the decoded reach, providing similar feedback to an exoskeleton, or possibly an FES interface. Before the decoders could be tested, training data was collected to train the models. Each experiment began with a set of training reaches in which EMG and kinematic data were collected. This involved the robot moving automatically along a straight-line trajectory to a set of nine targets spanning the monitor area. Each target appeared four times in random order. The subject was instructed to gently assist the reach as their hand was moved along the trajectory. EMGs were recorded (Figure 2) to quantify subject involvement and to train the decoding algorithms. We chose this method because we wanted control to be intuitive; it was important that the recorded EMGs corresponded as closely as possible to those a subject would naturally generate when attempting to make smooth reaching movements in our experimental setup. These same data were used to train all three decoders listed in Table 1, as we have described previously (Corbett et al., 2013a).

We presented subjects with a reaching task to evaluate decoding quality. For each trial a target randomly appeared on the monitor, 1 s before an auditory go cue. The goal was to place the stylus as close to the center of the target as possible. After the go cue, the reach was initiated when the square-root-transformed RMS value of any EMG channel increased above twice its level prior to the go cue. For the subject with no voluntary deltoid activation, the contralateral upper trapezius was also recorded to allow her to initiate reaches where she would not normally activate the ipsilateral muscle, by shrugging her left shoulder. However, this muscle was not included as a part of the decoder as it was not involved in the natural reach. Thus, while the subjects were unable to control the robot before the go cue, the reaches were self-paced in the sense that they could initiate them at their leisure after the cue. After initiation, the decoded velocity was used to control the robot's reach.

Upon initiation of a reach, the decoder was provided with the initial state vector including the robot's current position. When testing the KFT and mKFT, target estimates were also initialized in the state vector. In the case of the KFT, the actual location of the target center was provided. For the mKFT, the gaze data from the half-second period prior to initiation were used to estimate three potential targets with which to initialize a corresponding mixture component. The three-dimensional location of the eye gaze was calculated by projecting its direction onto the monitors. The first, middle and last samples were selected, and all other samples were assigned to a group according to which of the three was closest. The means of these three groups were used to initialize three KFTs in the mixture model and their priors were assigned proportionally to the number of samples in them. If the subject looked at multiple positions prior to reaching, including the target, the correct target would be accounted for in one of the mixture components.

Each target consisted of a green circle of 1 cm radius surrounded by five rings of various colors 1 cm thick. When the target was attained its color changed to that of the location corresponding to where the stylus touched. For a missed target, or if the reach timed out (after 10 s), the target turned red. For attaining the green circle the subject received a score of 10 points and for outer rings they received 9, 8, 7, 6, and 5 points. Feedback of the cumulative total of the most recent 10 reaches was displayed to increase motivation.

Each subject in Groups 1a and 2 performed an experiment for the interface with EMG alone (KF) and one for the models incorporating target information (KFT and mKFT). The order of these experiments was randomized across subjects. Due to difficulty obtaining an eye-tracking signal we were unable to test the mKFT with Subject 4, though he performed the KFT experiment. The KFT, with perfect target information, represented an idealized benchmark for the performance of a combined target and EMG decoder. After initial setup, each experiment began with the training protocol described above. In the KF experiments this was followed by between 10 and 30 practice reaches and 60 test reaches. For the experiments with target information, 60 KFT reaches were performed first. This was followed by eye-tracker calibration, up to 10 practice mKFT reaches and finally 60 test reaches with the mKFT. Eye-tracker calibration was checked periodically throughout the experiment and if found to be off, generally due to the headset shifting on the subject's head, we recalibrated the system and repeated any affected trials.

To put the decoder performance in context with the subjects' voluntary reaching abilities, we also asked them to attempt to reach each of the training targets while the HapticMaster was in “free mode,” supporting its own weight against gravity. This would differ from their unassisted reach abilities, as their arms were supported against gravity with the mobile arm support.

#### Assessing the influence of proprioceptive feedback on decoding performance

Many of the subjects could voluntarily activate sufficient EMG at the shoulder to make control with EMG alone viable. It was unclear whether this would be possible in a different decoding scenario such as an external robotic arm or computer-based interface, where their arm was not being moved in congruence with the decoder. The robot-assisted reaching task was providing these subjects with at least some natural proprioceptive information, and we wanted to establish how important a role this played in our results. Therefore, for the subjects who had more voluntary ability, we compared performance of the KF (with EMG alone) for both remote control of the robot and attached control as described in the previous experiment.

The protocol for attached control was exactly as described above. For remote control, the models were trained by the subjects attempting to mimic the movement of the robot as naturally as possible in the training reaches, without any physical attachment to the robot. In testing, subjects were free to move their arm as they wished while attempting to direct the robot to the targets. At least 20 practice reaches were performed before the testing reaches. This protocol meant that the conditions were compared using models that were trained on different data, a factor that we had previously found to have a small effect on performance in able-bodied subjects (Corbett et al., 2013b). However, we considered it more important to have consistency between training and testing for these subjects as, when unassisted, it may have been impossible for them to replicate the movements generated while attached to the robot in training. The order of the two conditions was randomized across subjects. To see whether any effect of removing feedback would hold when target information was included, we also tested the KFT remotely for two of the subjects.

### Analysis

We used two metrics to quantify performance in both experiments. The first was a measure of how accurately the target was achieved. This was quantified as the shortest distance between the stylus tip and the target center during the reach. As the target center had a 1 cm radius, any distance less than 1 cm would correspond to perfect task performance. The second measure was one of reach straightness, used to measure the efficiency of the generated movement. This was quantified as the path efficiency, the ratio of the cumulative distance of the reach to the straight-line distance. To put the results in context with the individual subjects' abilities, these measures were compared to their voluntary performance when the weight of the arm was supported by the passive mobile arm support. We then used the grouping system described above for statistical analyses. To compare the performance of the two decoders, and how this was affected by the subjects' impairments we used an analysis of variance (ANOVA) to look at the effect of the interaction of *algorithm* and *group* on the performance metrics, with *subject* as a random effect. Tukey tests were performed for *post-hoc* comparisons, and all statistical comparisons used a significance level of α = 0.05. To evaluate the effect of the proprioceptive feedback in the second experiment we compared the remote and attached conditions again using an ANOVA with *condition* as a fixed effect and *subject* as a random effect, with a Tukey *post-hoc*.

## Results

### Assessing the influence of impairment on decoder performance

**Figure 3:**
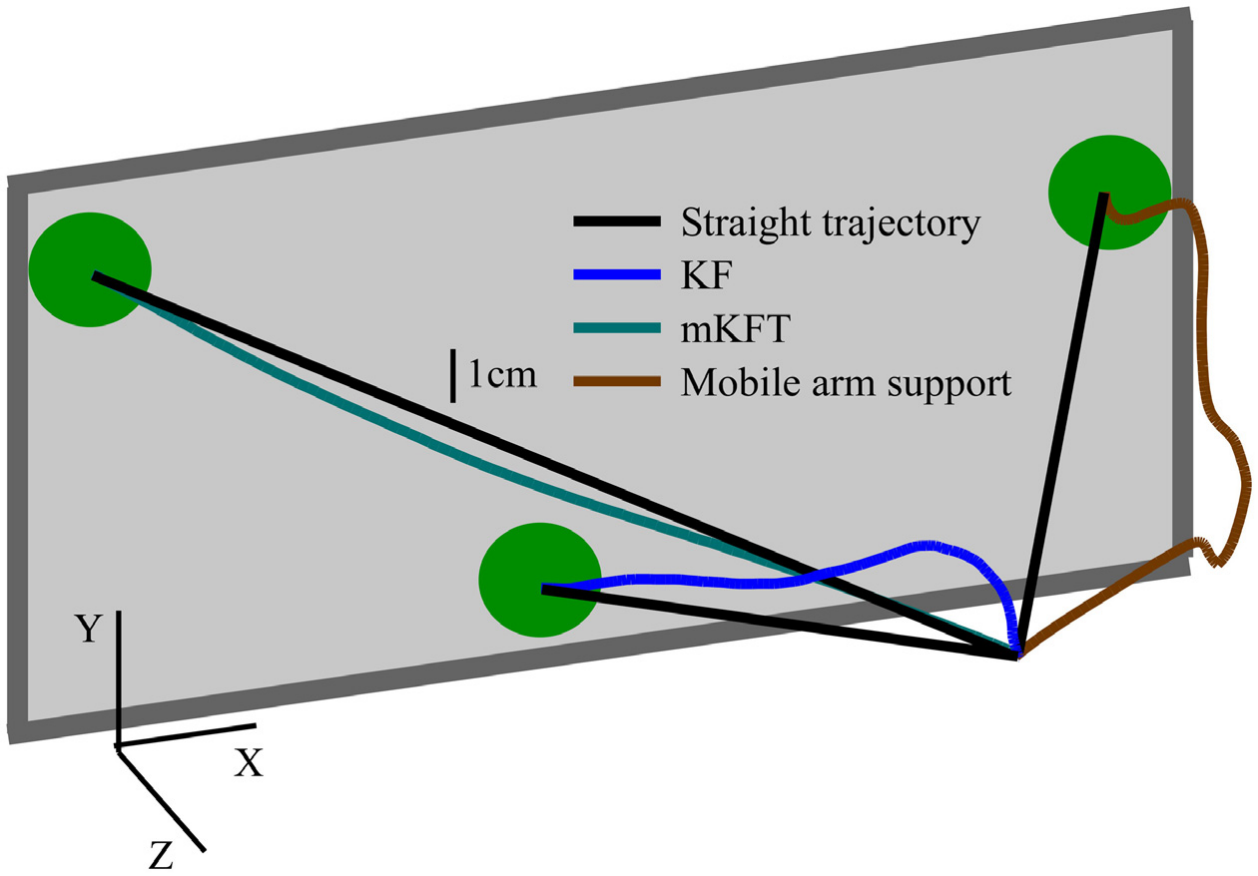
**Example reach trajectories under the various conditions with typical path efficiencies**. Kalman Filter (KF)—94.2%; mixture of KFTs (mKFT)—99.7%; Voluntary with mobile arm support providing support against gravity—67.5%. All reaches are by Subject 3. The monitor is for illustration purposes and is not to scale.

**Figure 4:**
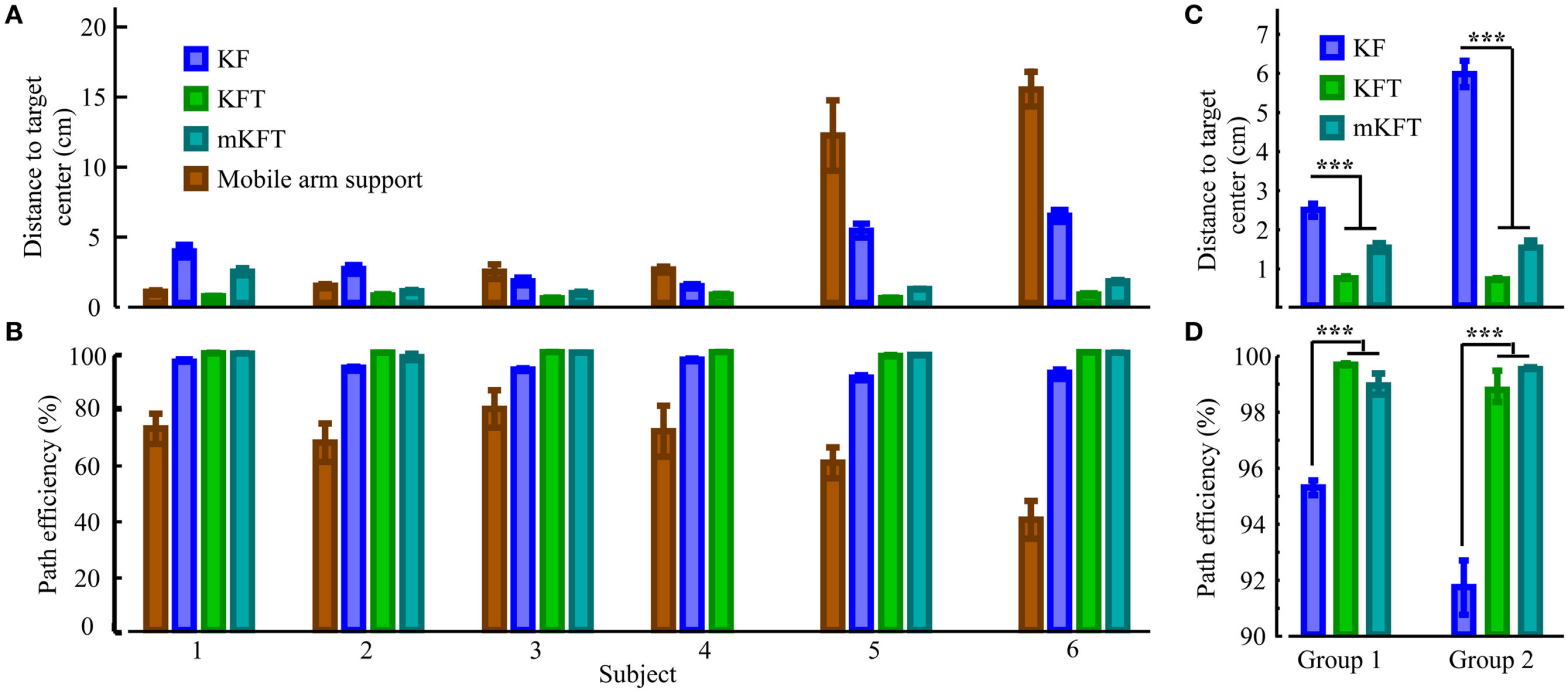
**Influence of subject impairment on decoder performance**. By subject in order of increasing impairment, along with perfect target information case (KFT) and volitional reach performance using mobile arm support: **(A)** Errors relative to the target, **(B)** Straightness of the reach. In groups: **(C)** Target errors, **(D)** Straightness. Statistically significant differences within groups are shown; ^***^p < 0.001.

As would naturally be expected, the subjects' voluntary ability to reach the targets when assisted by the mobile arm support depended on their impairment. In fact, some of the less impaired subjects could reach much of the target area with only this gravity assistance. However, the irregular shape of the trajectories, illustrated by one of the example reaches with typical path efficiencies (Figure 3), suggested that they did so with substantial difficulty, correcting for multiple errors over the course of the reach. The errors at the target measured when the subjects reached voluntarily with the mobile arm support increased with subject impairment level (Figure 4A). Path efficiencies did not follow a similarly fixed pattern but were clearly lowest for the two most impaired participants (Figure 4B). While some of the subjects were clearly unable to reach the targets with gravity support, others did better but left room for improvement, particularly in terms of reach straightness.

**Figure 5:**
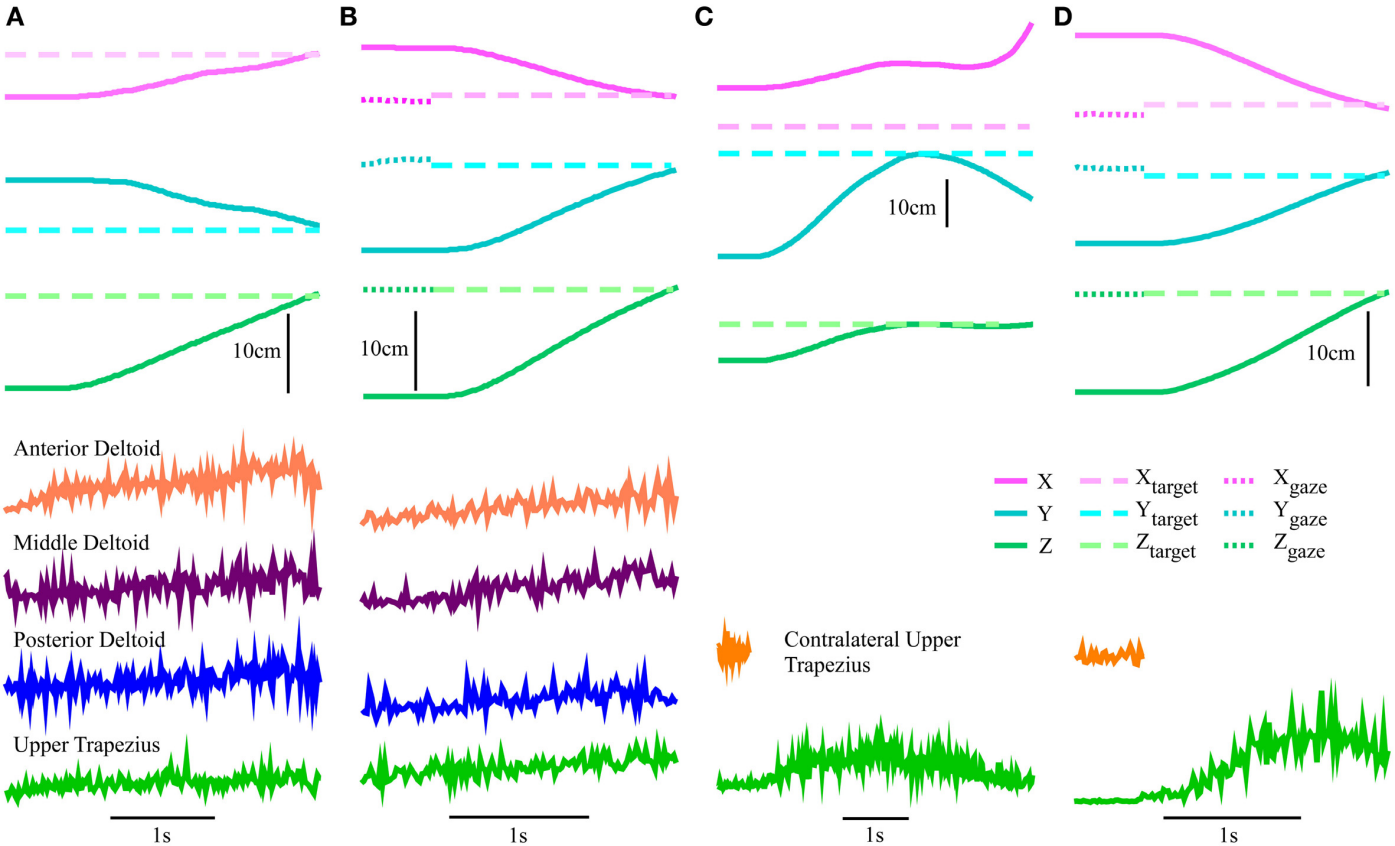
**Robot-assisted reach trajectories and the signal sources used to generate them**. Kinematics and square-root transformed root-mean-squared value (RMS) of EMG for an example reach. Subject 3 with **(A)** KF using EMG alone and **(B)** the mKFT, combining the gaze from the 0.5 s period before the reach initiation with EMG control; and Subject 6 with **(C)** KF using only the upper trapezius EMG and **(D)** mKFT.

The effectiveness of the KF decoder using EMG alone was also strongly dependent on the voluntary abilities of the subjects. Subjects from Group 1 could often guide the robot close to the target with their EMG signals (see example reach, Figure 5A), while those in Group 2 had greater difficulty (see example reach, Figure 5C). Subjects 1 and 2, the least impaired subjects, were in fact less accurate at the target with the KF than in the gravity assistance condition (Figure 4A). However, the decoder clearly provided improvements in reach straightness for these subjects (Figure 4B). For all other subjects the EMG-alone decoder provided improvements in both accuracy and straightness relative to the mobile arm support, and this was most pronounced for the two most impaired subjects (Figures 4A,B). Nonetheless, the accuracy and straightness of the reaches by the subjects in Group 2 were dramatically lower than those in Group 1 using the KF (Figures 4C,D). The EMG control allowed the more impaired subjects to reach toward the target-display monitor, but their accuracy was very poor.

The multimodal decoders were much more consistent across individuals and enabled accurate reaching for all subjects. Unsurprisingly, the distance to the target center was lowest for all subjects when perfect target information was available—there was very little variability for this condition. The KFT results were within the margin of error for perfect system performance, as the task required an accuracy of 1 cm for a perfect score (Figure 4). When gaze and EMG were combined (mKFT) the performance deteriorated slightly from that with perfect target information (*p* = 0.003), although this difference was not statistically significant when the subjects were separated into groups (*p* = 0.09 in Group 1, *p* = 0.39 in Group 2). For this decoder subjects took time to initiate the reach when they were ready—2.3 ± 0.7 s (mean ± SD) after the go cue—as the target estimates from the gaze position in the 0.5 s before reach initiation allowed them to make effective reaches straight toward the target (Figures 5B,D). Subject 1 was the least accurate of the group with the mKFT and was again less accurate than his performance with gravity support; he had some difficulty with the eye-tracking and thought he may have had a “lazy eye” (Figure 4A). All other subjects were consistently accurate with the mKFT. Both subject groups showed highly significant improvements between the mKFT and KF (*p* < 0.0001), and the difference between the two groups was minimal when the gaze was incorporated (*p* > 0.99). The incorporation of gaze allowed excellent target acquisition for all subjects, as would be expected with sufficiently accurate target estimates.

Reaches were also straighter for the models incorporating target information than for the one with EMG alone (Figure 4D). In both groups, the mKFT and KFT both averaged above 99% path efficiencies, and were not statistically different for either group (both *p* > 0.09). The KF, on the other hand, had average path efficiencies of approximately 95% for Group 1 and 92% for Group 2, which were significantly lower than the mKFT (both *p* < 0.001). This indicated that, while dramatically better than the gravity-supported reaches, the KF produced more errors in the trajectories that the users needed to correct for. Incorporating the target into the trajectory model generated more efficient, straight reaches.

Finally, to gain some insight into the subjects' EMG activation during mKFT control, we performed offline decoding using the KF algorithm, trained from the standard training data, of the reaches performed during mKFT control (KFT for Subject 4). We evaluated the accuracy of the reaches by calculating the *R*^2^ between the decoded reach and an “ideal” straight-line reach to the target, using the trajectory profile of the training reaches. In the subjects in Group 2 for whom EMG-alone control was clearly ineffective, there was no significant difference between the accuracy of the KF decoded offline and the online KF control (both *R*^2^ = 0.6, *p* > 0.9). The KF decoded offline was more accurate for the subjects in Group 1 (*R*^2^ = 0.7, *p* = 0.006), although substantially lower than online KF control in Group 1 (*R*^2^ = 0.9, *p* < 0.001). It is not surprising that without online feedback from the KF decoder the accuracy of the decoded reaches would be reduced. This result demonstrates that the users interact with each decoder differently, and can exploit the benefits of added target information during mKFT control. Nonetheless, the higher accuracy of the offline KF decoding in Group 1 suggests that for these subjects the EMG information can contribute to the decoding in mKFT control.

### Assessing the influence of proprioceptive feedback on decoding performance

**Figure 6:**
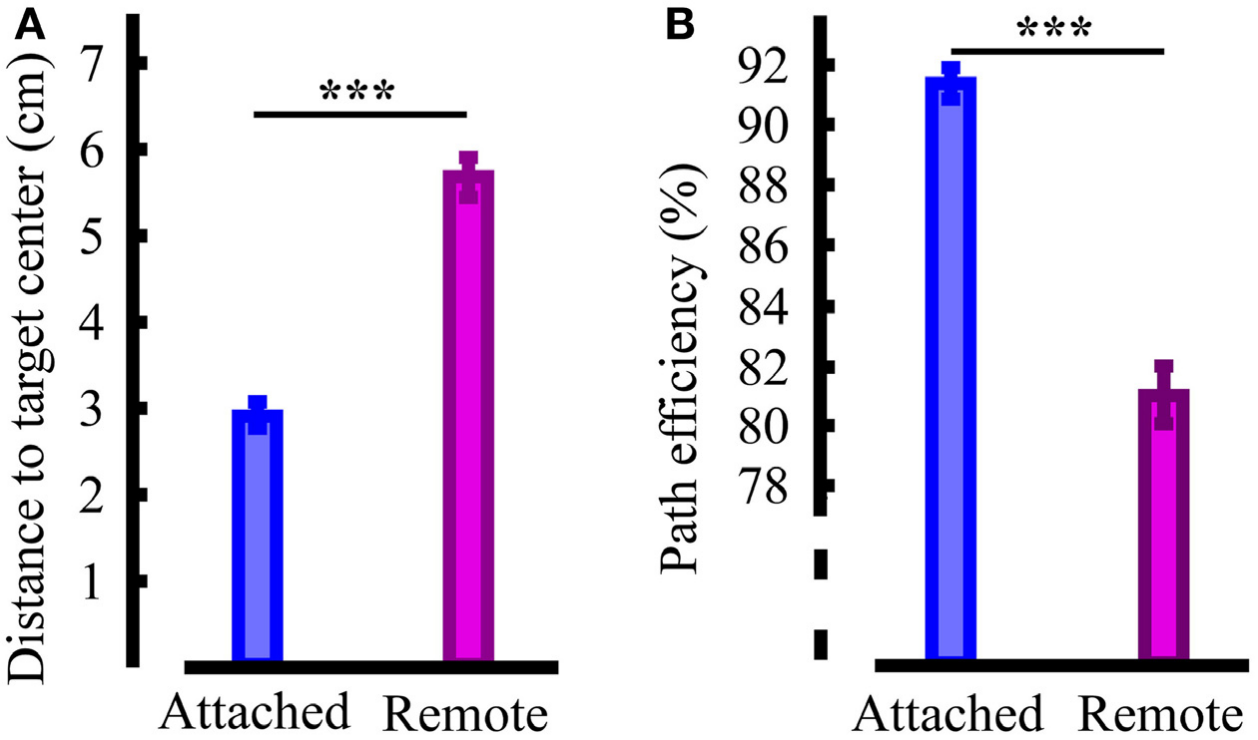
**Quantification of the influence of task related proprioceptive feedback**. **(A)** Reach accuracy; **(B)** Reach straightness. Statistically significant differences are shown; ^***^p < 0.001.

The above results show that while there was clearly an accuracy benefit to using the mKFT, for subjects in Group 1 reasonable control could be achieved using their EMG alone. We wanted to test the dependence of that performance on the natural proprioceptive feedback that was provided to the subjects by moving their arms. To do this, we compared the robot-assisted reaching task with the KF decoder to a remote control task where the subject had no mechanical link to the robot. We found that the remote performance was significantly less accurate than the attached condition, with the errors increasing from 3 to 5.5 cm (*p* < 0.001, Figure 6A). Path efficiencies were also reduced from 91% to an average of 81% (*p* < 0.001, Figure 6B). While it is possible remote control of the robot may have improved with further practice, this is unlikely as we did not see improvements over the course of the experiments, suggesting that the subjects were not learning further. Clearly, congruent proprioceptive feedback was a critical component of the interface for the subjects in Group 1, and reaches were less accurate and less straight without it.

To establish whether the importance of proprioceptive feedback extended to the decoder with target information, two subjects additionally performed remote control with the KFT. In this case errors were less than 1 cm, similar to the attached case above. The proprioceptive feedback was apparently critical only in the absence of target information, when the shoulder EMG alone guided the trajectory. With target information, accurate reaching was possible regardless of whether the subject's own arm or an external effector was being controlled.

## Discussion

Each person with an SCI will have a unique set of challenges associated with his/her injury, and identifying the best approach to assist with reaching involves careful consideration of a number of factors. In this study we examined the benefits of a multimodal approach to decoding, considering the impact of the various injury characteristics in the group of subjects. We also examined the effect of the proprioceptive feedback that subjects experienced when interacting with the reaching interface. Combining gaze and EMG enabled effective reaching for our participants, even for those who could volitionally activate an extremely limited set of muscles. With proprioceptive feedback of the trajectories, subjects with greater voluntary ability could also perform reaches with their EMGs alone. However, the reaches were less accurate and required the users to correct for errors over the course of the trajectories. When we removed the congruent proprioceptive information, subjects were unable to accurately control trajectories without additional information about the reach target. These results highlight the importance of providing proprioceptive feedback to neuroprothesis users where possible. Furthermore, they demonstrate the promise of incorporating target information, such as that from gaze, in the absence of sufficient feedback or trajectory-related physiological signals.

### Multimodal decoding and the influence of subject impairment

Enhancing the trajectory model with information about the reach target was extremely useful for generating accurate trajectories in our robot-assisted reaching task. Reassuringly, performance was in agreement with previous tests in able-bodied subjects using similar sets of EMGs (Corbett et al., 2013a). The incorporation of the gaze data consistently enabled more accurate reaching than control with EMG alone. Furthermore, the approach produced significant improvements in path efficiencies, indicating that the reaching required less effort from the user. In particular, gains in accuracy from incorporating gaze (mKFT) were dramatic for the most impaired participants in Group 2. While there was a large difference in performance between the groups with EMG alone, they were equally accurate when the gaze was incorporated.

Subjects adapted well to the multimodal interface, finding it accurate and easy to use. This was perhaps surprising for Subject 6 in particular who had not moved her arm volitionally in the 2 years since her injury. When asked how she felt about using the interface, she said it felt like she was naturally moving her arm. This is in contrast to performance with EMG alone, where both subjects from Group 2 had little to no control. They were enthusiastic about the mKFT, to which it was doubtlessly more intuitive and easier for them to adapt than the KF. The impressions from the less impaired subjects in Group 1 were more varied. As mentioned in the results, Subject 1 had some difficulty with the eye-tracking interface, which he attributed to a “lazy eye.” While the remaining subjects mostly found the mKFT easy to use, a few also enjoyed the challenge of the EMG-only decoder. For those who were particularly effective with the KF, the greater control over the trajectory was more interesting to them despite the fact that overall it was less accurate than the mKFT. The reduced effort that the multimodal decoder required of the user was also reflected in the reduced offline accuracy of the KF decoder on the mKFT reaches. This information could be useful for future attempts to find a balance between accuracy and allowing the user to use his/her capabilities as much as possible, allowing operation at the “challenge point” (Guadagnoli and Lee, 2004). Hence, even though most subjects in this study preferred the mKFT system, this feedback from the subjects emphasizes the importance of considering factors other than accuracy when determining the most appropriate system for a specific individual.

Assistive devices must be targeted to an individual's injury and, especially with support against gravity, some of the subjects in Group 1 could achieve remarkable performance even without a neuroprosthesis. A device controlling the entire movement of the arm as we have tested here would likely restrict their natural abilities and be unnecessary for these subjects. Nonetheless, many of the subjects would benefit from some assistance with reach, particularly with more distal movements. An assistive device working in seamless integration with their voluntary movements could potentially be enhanced with gaze information, possibly providing greatly improved ease of control. While the eye tracking system used in this study was for proof of concept and was not portable, there are more lightweight systems available at low cost that will be suitable for chronic use outside of the laboratory (Abbott and Faisal, 2012), and will require the development of robust calibration protocols. This multimodal approach could be useful in any situation involving selection between a small number of action candidates, and could also be adapted to a number of different signal sources. Cortical recordings have been used to decode both trajectory (Kim et al., 2008) and target information (Hatsopoulos et al., 2004), as have cortical surface potentials (Schalk et al., 2007; Pistohl et al., 2008; Flint et al., 2012) and non-invasive electroencephalogram and magnetoencephalogram-based systems (Hammon et al., 2008; Waldert et al., 2008). Furthermore, context about reach objectives could be found from scanning the environment and identifying potential targets. As it stands however, the developed interface is far more likely to be useful to people with high tetraplegia—injuries at C4 or above.

### The influence of proprioceptive feedback

For those less impaired subjects who had reasonable control with their EMG alone we found that the process of moving the arm in congruence with the decoder output was critical to its success, as removing this proprioceptive information resulted in a substantial drop in performance. During unimpaired motor control, people form a sense of their arm position in space through a combination of both visual and proprioceptive cues (Graziano, 1999). Both of these components play an important role in enabling people to reach toward targets in their workspace. However, with many assistive technologies users must rely on visual feedback alone. This is unfortunately unavoidable in many cases, as the most impaired individuals may lose all sense of proprioception. This work therefore highlights the importance of current efforts to restore proprioceptive information through artificial stimulation (London et al., 2008; Gilja et al., 2011; Berg et al., 2013), while emphasizing that it could be extremely effective where possible to provide neuroprosthesis users with natural proprioceptive information about the state of their device.

Some recent work has demonstrated that adding proprioceptive feedback is useful during BMI tasks. BMIs developed for stroke rehabilitation have greater therapeutic impact when the limb is passively moved by a prosthetic device (Birbaumer et al., 2008; Buch et al., 2008). Additionally, Ramos et al. found that providing proprioceptive feedback of hand opening and closing with an exoskeleton improved BMI performance in able-bodied subjects (Ramos-Murguialday et al., 2012). Similarly, in a closed-loop BMI based on intracortical recordings from non-human primates, Suminski et al. found that passively moving the arm improved performance of a 2-dimensional cursor control task (Suminski et al., 2010). Furthermore, Gaunt et al. tested providing proprioceptive feedback to a BMI user with complete paralysis but fully intact sensation. They found that in the absence of vision, moving her arm in congruence with a prosthetic arm improved control (Gaunt et al., 2013). While cortical recordings were not involved in the current study, these findings together highlight the parallels between general neuroprosthesis use, BMIs, and normal motor control.

In a decoding setting where a BMI or other neural interface is used to control an external device, the user must learn the new mapping or coordinate transformation that the decoder performs. It is critical that users are provided with effective feedback of these transformations, as trajectories are planned to be straight in visually perceived space (Flanagan and Rao, 1995). Therefore, if the goal of the BMI is to control a cursor on a screen, as in the studies mentioned above, the planning process involved may be different to that of a real reach. Providing proprioceptive cues may facilitate this planning process. As only the robot was being controlled in our paradigm, through EMG signals that are actively involved in the natural reach, we directly affected the control signals that the subjects could produce by moving their arms. This process may have provided them with greater awareness of the robotic system and facilitated more accurate and natural control, despite the fact that their sense of proprioception was impaired.

## Conclusions

With the amount of available signal sources and sensory information varying widely between potential users of neuroprostheses, the choice of assistive device and decoding approach must be considered separately for each individual's specific needs. Moving the arm through reaching movements clearly enables some users to get great benefit from proprioceptive information, and should be seriously considered for those who can take advantage of it. Unfortunately, this approach would be ineffective for people who have lost their sense of proprioception completely. Often, these same individuals have few signals they can volitionally activate that are related to a desired reach trajectory, making neuroprosthesis control a great challenge. A Bayesian approach taking account of the reach goal clearly has many advantages in improving reach accuracy, regardless of the feedback experienced by the user. Especially when the set of neural command signals is small, or the lack of proprioceptive feedback makes trajectory control difficult, gaze or other systems for identifying potential target locations could provide a significant improvement to a neuroprosthetic interface.

### Conflict of interest statement

The authors declare that the research was conducted in the absence of any commercial or financial relationships that could be construed as a potential conflict of interest.
